# Enhancing patient value efficiently: Medical history interviews create patient satisfaction and contribute to an improved quality of radiologic examinations

**DOI:** 10.1371/journal.pone.0203807

**Published:** 2018-09-26

**Authors:** Knud Nairz, Ingrid Böhm, Sebastiano Barbieri, Dieter Fiechter, Nicola Hošek, Johannes Heverhagen

**Affiliations:** Department of Diagnostic, Interventional, and Pediatric Radiology, Inselspital, University of Bern, Bern, Switzerland; Medical University Graz, AUSTRIA

## Abstract

Diagnostic radiology examinations are generally very efficient processes optimized for high throughput and for serving the needs of physicians. On the downside, streamlined examinations disrupt the personal relationship between diagnosticians and patients. The radiology associations RSNA and ACR consider low visibility of radiologists a threat to the profession. Therefore, they launched counter-acting initiatives that aim at increasing patient satisfaction by providing more personal attention and care, and by raising knowledge about the discipline. However, they did not formulate concrete instructions on how to integrate care by radiologists into the examination process while inhibiting the flow minimally. From an internal patient satisfaction survey, we have seen that patients rated satisfaction with care and attention by physicians relatively low, indicating that patients would welcome a possibility to communicate with radiologists. In a controlled experimental setting, we have then changed our process to include a short medical history interview. Thereby we could corroborate that lack of educated communication is the primary cause of diminished satisfaction and could establish that the duration of the encounter is not critical to achieving improvement. Importantly, the interview also helped to improve the quality of the examination. Thus, short medical history interviews are a very efficient way to increase value by maximizing patient satisfaction and examination quality. Our approach is easy to implement in other radiology clinics that are interested in becoming more patient-centered and in raising patient satisfaction.

## Introduction

Radiology examinations constitute an essential and indispensable part of modern medicine and accordingly cause a considerable fraction of health care costs [1,2]. Hence, the radiological discipline is not only driven by an urge to improve, but also by a constant economic pressure to rationalize the procedures and to further advance technically. Clearly, this development has been enormously successful with regard to efficiency, precision, and quality of the examinations and images. Yet, the associated emphasis on efficiency parameters—such as reporting time—is also associated with the downside that radiologists became barely visible to patients [3].

It is well established that communication between patients and physicians influences the quality of medical care [4]. Patient-doctor interactions are essential for creating a good inter-personal relationship, for fostering the exchange of information, and for facilitating the making of treatment-related decisions [5]. Importantly, an effective communication has been shown to have a beneficial influence on the outcome of a therapy, in part by reducing bad decisions or mistreatments, in part even by decreasing the likelihood of burnouts on the side of medical professionals [6]. Physicians may direct the emotions of patients by putting medical information in the context of their needs, expectations, and perceptions and thereby increase their approval and cooperation with medical care. In line with this, a successful communication is measurable by collecting data on the level of satisfaction with the medical services [7–10].

Given the importance of patient involvement in the medical care process, the lack of possibilities for patients to talk to the radiologists seems to be detrimental. The radiological associations ACR and RSNA consider this shortcoming a potential threat to the profession and therefore promote a more patient-centered radiology [11, 12]. Hence, they launched several counter-acting initiatives and campaigns, such as “Face of Radiology”, “Five Patients per Day”, or “Radiology Cares” to enhance patient-doctor interactions and communication [13–15]. It is not clear how successful the campaigns were in the long run, because ACR and RSNA do not provide exact instructions on how to implement such drastic changes in the streamlined examination process and how to measure its success.

For internal purposes, we conducted a patient satisfaction survey and became aware that patients rated attention and care by physicians low when they had no contact with radiologists. We tried to address that problem in a controlled experimental setting by imposing a patient-doctor interaction that was minimally interruptive for radiologists. This change in the diagnostic radiology process raised patient satisfaction to a level that was comparable to much longer interactions and incidentally improved the quality of radiologic examinations. Thus, we describe an approach to reconcile the need of patients to communicate with the need of process optimization in diagnostic radiology and provide a blueprint for a patient-centered and efficient radiology.

## Materials and methods

### Ethics

We performed the study in accordance with the policy of the Institutional Review Board (Kantonale Ethikkomission) of the Kanton of Bern (Switzerland). As we documented neither the patients’ names, birth dates, nor their sex or other parameters that could identify the individual patient, an informed signed consent was waived by KEK-Bern (Reg-2016-00729).

### Survey design and logistics

For internal organizational purposes, we wanted to conduct a patient survey in order to get objective data on what is going well and where there is room for improvement in our radiologic examination processes, putting emphasis on magnetic resonance imaging. The composition of the survey was based on the prime consideration that patients want to be treated competently, quickly, friendly, and caringly [3, 12, 16]. Therefore, we centered the questions on their needs, perceptions, experiences, and recommendations.

In order to be all-embracing and meaningful, the survey was intended to cover all steps of the examination that are visible to patients. On the other hand, being considerate of the target group we also put emphasis on a clear design according to the corporate design guidelines of our hospital, good readability, and big font size, thus limiting the amount of questions (S2 Fig). Thereby we abstained from obtaining any personal data and from polling the scheduling process, which is usually handled by referring clinics. We opted for a sexpartite scale ranging from maximally 6 points to minimally 1 point. This scale bears some resemblance to the Swiss school grading system, where marks equal or greater than 4 are positive and smaller than 4 negative.

Overall, we formulated two questions about general satisfaction and fifteen questions about relevant steps of the radiologic examination process (Table 1), accounting for examples in the literature and a routinely conducted hospital-wide survey from the Swiss National Association for Quality Development in Hospitals and Clinics [17, 18]. As our survey was originally intended to cover MRI-exams only, one question was MRI-specific. However, we then decided to enroll patients who were admitted for other modalities as well and consequently added a multiple-choice question about the examination modality. Similarly, we included choice questions about the type of stay (in- or out-patient), and about waiting time (Table 1). Thereby, we queried all patients with a question specific for MRI-exams (question no. 5, Table 1) and along the way created an excellent internal control about the feedback quality (Results ‘Response rates are informative’).

**Table 1:** Questionnaire and consolidated results of the patient satisfaction survey in three consecutive years. Data are expressed as percentages of answers with positive grading, i.e. as the relative occurrence of grades 6, 5, and 4, (including the 95% confidence interval). Responsiveness (times answered) is given in absolute numbers and in percentages. This rate appears low in question 5 about an MRI-specific question, in question 6 about waiting time, and in question 9 and 13 about contact with radiologists (in bold).

Question no.	Question	% positive grading (6, 5, 4) of answered questions (95% Wilson confidence interval)	times answered (%)
1	How was your scan performed?		
	Out-patient		761 (75.9%)
	In-patient		215 (21.4%)
	left blank		27 (2.7%)
2	Which scan was performed on you?		
	MRI		438 (43.7%)
	CT		140 (14.0%)
	Angiography		7 (0.7%)
	Mammography		0
	X-ray		195 (19.4%)
	Fluoroscopy		17 (1.7%)
	Ultrasound		183 (18.2%)
	several		20 (2.0%)
	left blank		3 (0.3%)
3	How long was your waiting time?		
	Less than 15 min		811 (80.9%)
	15–30 min		123 (12.3%)
	more than 30 min		46 (4.6%)
	left blank		23 (2.3%)
4	How have you been welcomed?		
4a	a) Friendliness	99.6% (99.0–99.8)	999 (99.6%)
4b	b) Competence	99.6% (98.9–99.8)	945 (94.2%)
5	Have you been adequately informed about the health questionnaire?	96.8% (95.4–97. 8)	840 **(83.7%)**
6	Have you been informed about the waiting time?	**89.6%** (87.4–91.4)	891 **(88.8%)**
7	How did you perceive the waiting time?	**92.7%** (90.9–94.2)	948 (94.5%)
8	How have you been looked after by the radiologic technologists?		
8a	a) Friendliness	99.6% (99.0–99.8)	998 (99.5%)
8b	b) Competence	99.7% (99.1–99.9)	940 (93.7%)
9	Have you had contact with a physician at this clinic, either before, during, or after the radiology scan?	**57.4%** (54.1–60.5)	910 **(90.7%)**
10	Was consideration shown for your state before and during the radiology scan?	98.3% (97.3–99.0)	965 (96.2%)
11	How did you perceive the radiology scan?	**90.4%** (88.4–92.0)	986 (98.3%)
12	Were you well cared for and dismissed after the scan?	98.9% (98.1–99.4)	983 (98.0%)
13	How do you appraise the care by physicians?	**91.0%** (88.8–92.7)	841 **(83.8%)**
14	How do you judge the radiological service as a whole?	99.0% (98.1–99.5)	981 (97.9%)
15	Can you recommend the Radiological Institute to your friends and relatives?	99.1% (98.3–99.5)	989 (98.6%)
			Total: 1003 (100%)

An accompanying message of greeting asked the patients to answer the questionnaire truthfully and informed that the answers would not have any influence on the medical treatment whatsoever (S2 Fig). After the examinations, our technologists gave a questionnaire to all patients who were physically and mentally fit and asked them to complete the form. Further information or help with completion was only given upon request.

The questionnaire was validated in 2013 when a big internal survey was conducted within 5 weeks comprising about 600 patients, who had been examined by 6 different modalities (Table 2). The collected data seemed to be meaningful and conclusive, as e.g. the perception of the radiologic examination depended on the modality or the general satisfaction was correlated with the waiting time.

**Table 2:** Number of surveys, modalities, and type of exams conducted and the number of surveys uniformly grading top grade 6.

	validation	1. repetition	2. repetition	sum
** type**				
out-patients	399	182	180	761
in-patients	191	0	24	215
sum	606	182	215	967
(left blank)	(16)		(11)	(27)
sum				1003
** modalities**				
MRI	162	182	94	438
CT	140	0	0	140
Angiography	7	0	0	7
Mammography	0	0	0	0
X-ray	195	0	0	195
Fluoroscopy	17	0	0	17
Ultrasound	62	0	121	183
several	20	0	0	20
(left blank)				(3)
sum	603	182	215	1003
**modalities+type**				
MRI, out-patients	154	182	93	429
MRI, in-patients	8	0	1	9
US, out-patients	45	0	87	132
US, inpatients	16	0	23	39
MRI, + medical interview			47	
MRI,—medical interview			47	
**questionnaires uniformly graded “6”**				
all questions answered	37	14	22	73
some questions left blank	85	22	34	141
(proportion)				(21.3%)

An apparent correlation of satisfaction with care by physicians and the visibility of radiologists led us to hypothesize that satisfaction of patients with their attending radiologists is not only based on a basic trust in the medical expertise, but also on the possibility to communicate about their medical state. However, it was not clear to what extent a radiologist would have to engage in patient care in order to reach levels that are associated with a 30-minutes (ultrasound-) exam. We also were undecided whether it would be better to communicate a provisional diagnosis after the exam or discuss the medical history prior to the exam. Organizational constraints led to the plan to introduce a medical history interview for MRI-patients prior to the exam. One year later in 2014, we implemented this process change. The survey was conducted concomitantly with the implementation of interviews for about one month and was performed as a prospective, observational, and mono-center study.

In 2015, we repeated the survey again and questioned both MRI- and ultrasound-patients throughout the year (timeline shown in S1 Fig). Additionally, some ultrasound patients, who responded with grade 5 or lower to question 9, had a personal interview about their motivation based on an additional structured questionnaire (results in S4 Table).

Personal interaction with radiologists was arranged in the context of a patient medical history interview that usually did not last longer than three minutes and thus was little disruptive for reporting. In support of the medical history interview, radiologists received a form sheet, which provided structure for the interview and included the following questions:

Which body part/organ shall be examined?

Which side? (left/right)

Since when does the ailment exist?

[Place for] Description of the ailment/sketch

Besides, a check box about the need to change the original MRI protocol was introduced. The percentage of such adaptations due to insights from the interviews was taken as a quality measure (Table 8).

### Study population

The study was conducted at the Institute of Diagnostic Radiology at the Inselspital Bern, the largest radiology practice in Switzerland. Inclusion criteria were non-restrictive: the patients had to be adult, to be willing to participate, and to be able to read and to understand the questionnaire. Participants were both in- and out-patients and examined by either MRI, CT, ultrasound, conventional X-ray, angiography, or fluoroscopy. The present study summarizes results from three rounds/years of interrogations conducted between 2013 and 2015 (Tables 1 and 2, S1 Fig).

### Statistics

Survey data are expressed as percentages of answers with positive grading, i.e. as the relative occurrence of grades 6, 5, and 4. A 95% Wilson confidence interval was determined for those values. For each question, grades were then categorized into positive (6, 5, 4) and negative (3, 2, 1) classes. Besides, we classified responses as either *left blank* or *answered*. Potential differences in the distribution of those classes were tested by chi-square statistics and expressed as derived P-values. Statistical significance was set at P values <0.01. The calculations were performed with the statistics program STATA2, version 12.1 (StataCorp LLC, Texas, USA).

## Results

### Questionnaire and rating

We designed an anonymous patient survey that depicted the most relevant steps of the radiological exam process from the patient’s perspective (Table 1, Materials and Methods).

The survey consisted of total 17 questions specifically aimed at friendliness and competence of the personnel (four questions), proper information (three questions), care and empathy (two questions), and waiting times (three questions) (see Materials and Methods). Besides, we asked about the overall satisfaction with our service and about a potential recommendation of our service to family and friends. A further question targeted the problems of the apparently blurred occupational profile of radiologic technologists and radiologists, as well as of the public image of diagnostic radiologists, who often appear invisible to patients (question 9). The latter topic was also weaved in a question about care and empathy (question 13). Finally, we polled a subjective statement about the personal well-being during the radiology exam where we anticipated dependencies on the chosen modality (question 11).

As we did not have empirical data about how informed our patients are about the occupational profile of technologists and radiologists and about their knowledge of radiologists being physicians, we were especially interested in questions 9 and 13. There we asked whether they have had contact with *physicians* (rather than *radiologists*) and about the quality of care that they have received by *physicians*.

Additionally, we queried three general subjects by multiple choice to record the modality, the type of stay, and the perceived absolute waiting time (questions 1 to 3).

We asked patients to grade akin to the Swiss school grading system on a sexpartite scale from maximally 6 points to minimally 1 point; they were also free not to answer a question. In the Swiss school system, grades equal or greater than 4 are positive and grades smaller than 4 are negative.

In the first year, 93% of the questionnaires handed out were returned, in the second year 91%, and in the third year 94% (S1 Fig).

As we conducted the survey anonymously, we could not link the obtained results to age groups, sex, or diseases.

The total study population comprised of 761 out- and 215 in-patients and the status was unknown in 27 participants. Most patients underwent MRI-scans (n = 438), followed by conventional X-ray scans (n = 195), and ultrasound examinations (n = 183) (Tables 1 and 2). We validated the questionnaire on a population of 606 patients (Table 1, S3 Table), derived our conclusions (see Materials and Methods), and repeated the survey twice with 182 and 215 patients, respectively, after the implementation of medical history interviews (S1 Fig).

### Survey grading

**Fig 1:**
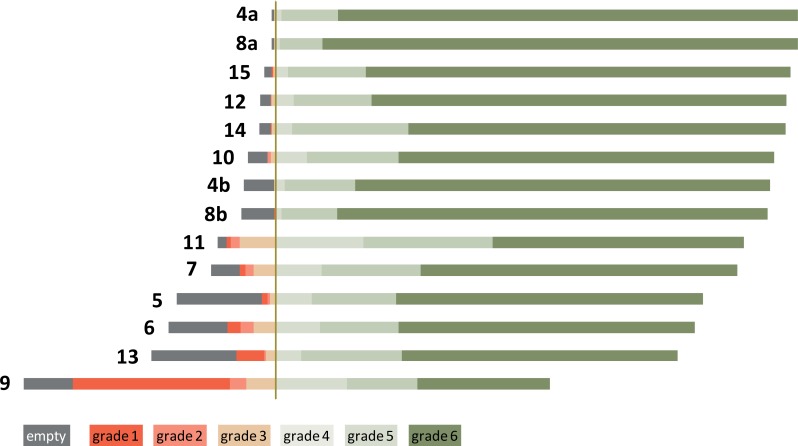
Combined distribution of grades and unanswered questions (empty) of the complete survey dataset. Numbers to the left of the bars indicate questions that are exemplified in Table 1. The vertical line separates positive (grades 6, 5, 4) from negative (grades 3, 2, 1) appraisements.

Overall, patients graded very approvingly such that survey results were highly skewed to grade 6 or 5 (Fig 1, Table 1). The best rating questions were “Friendliness” of both administrative staff and radiologic technologists, and the lowest score was given for question 9 (contact with the radiologist).

21.3% of the participants rated uniformly with the highest grade “6”, one-third of them answering all questions (Table 2).

### Data validation

Usually, patient satisfaction is highly dependent on the waiting time [19]. To test whether this holds true in our data set as well, we analyzed the general satisfaction with our services (question 14) and the potential recommendation of our services (question 15) in groups stratified for short (less than 15 minutes) and long (more than 30 minutes) waiting times. The distribution of grades was significantly skewed towards more positive values in the data set of patients experiencing short waiting times Table 3, S1 Table).

**Table 3:** Pairwise comparison of overall satisfaction and recommendation of our radiology services dependent on subjective waiting times (less than 15 or more than 30 minutes). Combined survey data are presented. Data are expressed as the percentage of positive grading including a 95% confidence interval. Significances are calculated for the distribution of positive (6, 5, 4) versus negative (3, 2, 1) grading. Significances at the 99% confidence level or higher are marked in bold. For exact phrasing of questions, refer to Table 1. More detailed data are shown in S1 Table.

	positive grading (6, 5, 4) in % of answered questions (95% Wilson confidence interval)		chi-square P value of positive (6,5,4) vs. negative (3,2,1) grading
question	>30 min	<15 min	
14 (service overall)	95.7% (85.5–98.8)	99.4% (98.5–99.7)	**0.007**
15 (recommendation)	93.5% (82.5–97.8)	99.4% (98.5–99.7)	**<0.001**
numbers	(46)	(811)	

Likewise, it is conceivable that satisfaction with the hospital in general would have an impact on the satisfaction with radiology. E.g., it has been shown that the objective physical environment affects satisfaction levels in in-patients and outpatients divergently [20]. We therefore stratified our data set for in-patients and outpatients and explored whether the general satisfaction with our services (question 14) and the potential recommendation of our services (question 15) would be answered differently. We did not observe any statistic deviation in general satisfaction in the two groups and conclude that potentially divergent satisfaction levels with the hospital do not influence our data (Table 4, S2 Table).

**Table 4:** Pairwise comparison of overall satisfaction and recommendation of our radiology services dependent on the type of stay (in-patient or outpatient). Combined survey data are presented. Data are expressed as the percentage of positive grading including a 95% confidence interval. Significances are calculated for the distribution of positive (6, 5, 4) versus negative (3, 2, 1) grading. Significances at the 99% confidence level or higher are marked in bold. For exact phrasing of questions, refer to Table 1. Data that are more detailed are shown in S2 Table.

	positive grading (6, 5, 4) in % of answered questions (95% Wilson confidence interval)		chi-square P value of positive (6,5,4) vs. negative (3,2,1) grading
question	in-patient	out-patient	
14 (service overall)	99.0% (96.6–99.7)	99.2% (98.3–99.6)	0.822
15 (recommendation)	99.5% (97.4–99.9)	99.2% (98.3–99.6)	0.613
numbers	(215)	(761)	

### Factors influencing patient satisfaction

A closer analysis of our first-year survey results revealed in part significant differences in the average satisfaction between ultrasound and MRI patients (Table 5).

**Table 5:** Original, first-year survey responses by ultrasound and MRI patients. Data are expressed as the percentage of positive grading including a 95% confidence interval and as the percentage of answered questions. Significances are calculated for the distribution of positive (6, 5, 4) versus negative (3, 2, 1) grading and for answered versus left blank questions. Significances at the 99% confidence level or higher are marked in bold, significance at the 95% level in italic. Ultrasound patients experiencing contact with radiologists have a significantly higher response rate at questions 9 and 13. On the other hand, they very frequently did not respond to question 5 about an MRI safety questionnaire. For exact phrasing of questions, refer to Table 1. Detailed data for the other questions are shown in S3 Table.

	positive grading (6, 5, 4) in % of answered questions (95% Wilson confidence interval)			left blank in % of number of surveys		
question	MRI	Ultrasound	P-value (chi-square test)	MRI	Ultrasound	P-values (chi-square test)
5 (safety questionnaire)	96.8% (92.7–98.6)	93.5% (82.5–97.8)	0.311	3.7%	25.8%	**<0.001**
9 (contact with physician	28.0% (21.4–35.7)	83.9% (72.8–91.0)	**<0.001**	7.4%	0%	*0*.*028*
11 (pleasantness)	83.3% (76.7–88.4)	96.8% (89.0–99.1)	**0.007**	3.7%	0%	0.125
13 (care by physician)	86.2% (79.9–91.6)	95.1% (86.5–98.3)	0.083	20.4%	1.6%	**<0.001**
number	(162)	(62)				

**Fig 2:**
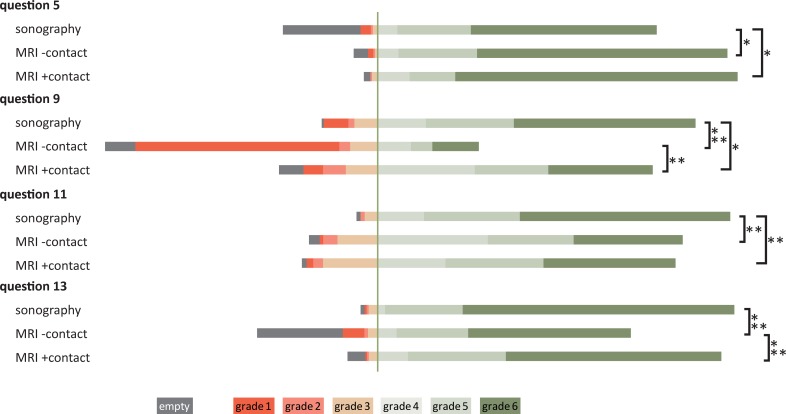
Grading and response rates by ultrasound patients (sonography) and MRI patients with or without contact with radiologists (MRI +contact, MRI–contact, respectively). Combined results from all three years are shown. Brackets marked with one asterisk denote significantly different response rates, brackets marked with two asterisks denote significantly different grading. The vertical line separates positive (grades 6, 5, 4) from negative (grades 3, 2, 1) appraisements. For exact phrasing of questions, refer to Table 1. Detailed data are shown in S8 Table.

This result was to be expected for two questions: 1) question 5 relating to an MRI-safety questionnaire handed out to MRI patients only and 2) question 11 concerning the pleasantness of the exam, which is perceived inconvenient by many MRI patients. Moreover, ultrasound patients significantly rated contact with and care by physicians (questions 9 and 13) better than patients who underwent MRI (Table 5 and Fig 2). At our department, radiologists perform ultrasound exams that usually last for about 30 minutes. MRI patients, on the other hand, had only scarce contact with radiologists, because technologists usually perform venous access for MRI contrast agent administration and native examination usually did not involve physicians, either.

We therefore hypothesized that satisfaction with physicians was dependent on their presence and visibility to patients.

### Ambiguous phrasing causes data variability

As our patients judged very affirming and had an excellent general impression of our services (questions 14 and 15), we were wondering about the apparent negative rating of question 9 enquiring contact with physicians (Table 1, Fig 1). We hypothesized that the outcome was due to a potentially ambiguous phrasing of the interrogation, which has a binary outcome (yes or no). Yet we maintained the sexpartite scale (from *very much* to *none at all)* for esthetical reasons. We scrutinized that statement and additionally interrogated in a structured manner 38 ultrasound patients (who had continuous contact with radiologists) and asked the 22 patients rating lower than 6 about their motivation (S4 Table). Each 10 of those either were confused or gave a quantitative estimation between 1 and 6 of the number of doctors they have seen. Diverging interpretations of this question caused a quantitatively more pronounced outcome as compared to question 13, but the qualitative predications are the same as will be shown below.

### Response rates differ

We noticed that patients left some questions unanswered (Table 1, Table 5) and wondered whether response rates would be informative to some extent.

We, therefore, stratified our sample and classified responses as being answered or being void. Potential deviations in the distributions were assessed by chi-square statistics (Materials and Methods). Especially, we were interested in whether response rates correspond to peculiarities of the radiology exam process. For example, question 5 was specifically created for MRI patients and was answered by more than 96% of the MRI patients, but by less than 75% of the ultrasound patients who did not get the enquired MRI safety questionnaire. There was also a significant difference in the response rate for questions about contact with and care by physicians: e.g., MRI patients without contact had lower response rates than ultrasound patients (Table 5). We hypothesize that non-responsiveness is an indicator of uncertainty about the interrogation, presumably because patients felt unable to rate a service, which they have not experienced personally.

### Medical interviews as an efficient promoter of patient satisfaction

The survey data suggested to us that patients would appreciate a personal contact with a radiologist. Based on experience and processual needs we decided that a patient-to-radiologist contact should be best in the context of a short interview prior to the exam. A medical interview would create more patient value than a mere “meet and greet” introduction of the examiner as suggested by the *Face of Radiology* and *5 Patients per Day* campaigns [14, 15]. The experiment was restricted to MRI patients only.

Concomitant to the introduction of interviews by radiologists we surveyed our MRI-patients using the same questionnaire (Materials and Methods). In the first round, 86% of 182 surveyed MRI-patients had short medical interviews prior to the scan. In the consecutive year, we surveyed a group of 47 MRI patients who had an interview and an even group without interview (Table 2, S1 Fig). The interviews usually lasted for about 3 minutes and were protocolled by the examiner (Materials and Methods). MRI patients with an interview rated most of the questions statistically indistinguishable to the MRI-patients in the original survey, indicating solid sampling and a stable examination process (Table 6, S5 Table). However, the change in the process was clearly reflected in the survey questions 9 and 13 concerning contact with and care by radiologists (Table 6).

**Table 6:** MRI patients in the original survey (without interview) and in the survey conducted when the interviews were introduced. Data are expressed as the percentage of positive grading including a 95% confidence interval and as the percentage of answered questions. Significances were calculated for the distribution of positive (6, 5, 4) versus negative (3, 2, 1) grading and for answered versus left blank questions. Significances at the 99% confidence level or higher are marked in bold. Patients experiencing contact with a radiologist rate questions 9 and 13 significantly higher and respond to question 13 more often. For exact phrasing of questions, refer to Table 1. Detailed data for the other questions are shown in S5 Table.

	positive grading (6, 5, 4) in % of answered questions (95% Wilson confidence interval)			left blank		
question	without patient interview	with patient interview	P-values (chi-square test)	without patient interview	with patient interview	P-values (chi-square test)
5 (safety questionnaire)	96.8% (92.7–98.6)	97.8% (94.4–99.1)	0.590	3.7%	2.2%	0.407
9 (contact with physician	28.0% (21.4–35.7)	80.6% (74.0–85.8)	**<0.001**	7.4%	6.6%	0.767
11 (pleasantness)	83.3 (76.7–88.4)	79.9% (73.4–85.1)	0.418	3.7%	1.6%	0.233
13 (care by physician)	86.8 (79.9–91.6)	96.6 (92.8–98.4)	**0.001**	20.4%	3.3%	**<0.001**
number	(154)	(182)				

Patients rated care by physicians significantly better when they had an interview and the increase in grading was even more accentuated in question 9 about contact with physicians as discussed before (Fig 2 and Table 6). Strikingly, patient satisfaction with radiologists was statistically indistinguishable between ultrasound patients (surveyed in the year before) and MRI-patients who had a medical interview (Fig 2 and Table 7). We also stratified our complete dataset in patients with an interview (ultrasound, MRI upon interview introduction, first MRI-group in second repetition) and in patients without contact with physicians (X-ray, CT, MRI during original survey, second MRI-group in second repetition). Both the response rates and the satisfaction with radiologists were significantly higher amongst patients who had a personal contact (S7 Table).

**Table 7:** MRI patients upon introduction of the medical interview and ultrasound patients (original survey). Positive grading and response by ultrasound patients and MRI patients in the consecutive year (who had contact with physicians). Data are expressed as the percentage of positive grading including a 95% confidence interval and as the percentage of answered questions. Significances at the 99% confidence level or higher are marked in bold, significance at the 95% level in italic. Significant differences in the responses are due to the modality (questions 5 and 11). However, satisfaction with radiologists is indistinguishable (questions 9 and 13). For exact phrasing of questions, refer to Table 1. Detailed data for the other questions are shown in S6 Table.

	positive grading (6, 5, 4) in % of answered questions (95% Wilson confidence interval)			left blank		
question	MRI	Ultrasound	P values (chi-square test)	MRI	Ultrasound	P-values (chi-square test)
5 (safety questionnaire)	97.8% (94.4–99.1)	93.5% (82.508–97.8)	0.137	2.2%	25.8%	**<0.001**
9 (contact with physician	80.6% (74.0–85.8)	83.9% (72.8–91.0)	0.569	6.6%	0%	*0*.*038*
11 (pleasantness)	79.9% (73.4–85.1)	96.8% (89.0–99.1)	**0.002**	1.6%	0%	0.309
13 (care by physician)	96.6 (92.8–98.4)	95.1% (86.5–98.3)	0.595	3.3%	1.6%	0.493
number	(182)	(62)				

### Medical interviews reduce MRI protocoling errors

If the appearance of a radiologist leaves such an impression on the patients, one might argue that a simple “meet and greet” would have a similar effect on patient satisfaction and thereby would be even more effective. In order to scrutinize that possibility, we conducted an analysis of 2700 medical history protocols of MRI-patients authored from introduction of interviews upon the end of surveying.

Overall, we found 27 (1%) cases where the originally specified MRI examination protocol (which usually is determined the day prior to the exam) had to be changed due to information from the medical interview. The reasons for the alterations have been extracted from the respective patient records and are listed in Table 8.

**Table 8:** Incidence and reasons for medical history-associated changes of MRI-examination protocols.

	Reason for anamnesis related changes	number
Medical history analyzed		2673
Change of examination protocol		27 (1.0%)
	other body region incl. wrong side	5
	expanded body region	3
	narrowed body region	2
	waiver of contrast agent	3
	inclusion of contrast agent	2
	other MRI protocol	8
	other MRI protocol due to scientific study	1
	n.d.	3

Presumably, protocol changes related to choosing another body region would not have remained undetected by our radiology technologists, but most of the other adaptations required medical competence.

## Discussion

Possibly, radiology is the medical discipline most affected by technological advances [1]. Technological improvements in combination with an ever-increasing workload, shortage of radiologists, and necessity of uninterrupted and concentrated work may be the major factors that led to an almost complete separation of patients from their radiology diagnosticians [2, 3, 21]. This development has been recognized as a potential threat and challenge to the profession by the medical associations ACR and RSNA. In order to counter these tendencies, they have therefore launched campaigns like *Radiology Cares*, *Patient-Centered Radiology*, *Face of Radiology*, *Five Patients a Day*, or *Imaging 3*.*0* [3, 12–15]. E.g., the RSNA *Radiology Cares* initiative stresses patient experience and advocates a “meet and greet” prior to the exam and a discussion of results after the exam. It is conceivable that the campaign has not been rigorously implemented, because exact instructions on how to introduce such drastic changes in the examination process and outcome measurements about the respective impact and usefulness of such measures were not given.

To the best of our knowledge, this is the first systematic study to confirm the importance of a medical interview prior to diagnostic radiology exams for both patient satisfaction and examination quality. Others have addressed the interesting questions whether radiologists should communicate the findings of the examination and their impressions directly to patients and thereby communicate with their patients after the exam [21–23] or have demonstrated the superiority of a radiologist versus a health questionnaire to obtain relevant medical information prior to an MRI exam [24].

When we started with an ordinary patient satisfaction survey in our diagnostic radiology department, we first noticed that patients rated contact with and care by physicians relatively low when compared to calibrator questions that allowed us to establish base levels in grading:

Interrogation about the subjectively perceived examination comfort or about waiting time defined the baseline of patient satisfaction, even though there was a very positive prevailing mood in the replies with 21% of the responders giving only maximal scores. It was therefore unexpected that the question about contact to physicians was skewed towards seemingly negative values. A sample testing then revealed that patients were in part confused about the apparently ambiguously phrased question and gave a quantitative assessment rather than a grade.

We further noticed that a majority of patients answered a question addressing a MRI-safety questionnaire, even when they were examined by another modality and did not receive a questionnaire. A closer analysis revealed that although a majority of our patients responded to every question, the response rate about the MRI-questionnaire was only high for MRI-patients and significantly lower amongst other patients (Tables 5 and 7). Similarly, MRI-patients had low response rates about the care of physicians (no. 13) when they had no contact with physicians (Tables 5 and 6). We hypothesize that non-responders refrained from answering, because they did not relate the question to their experience. Therefore, the response rate may be seen as an indicator that is correlated with the relevance of a question to the patient.

In summary, we concluded that both the rating and the withheld answers indicate that patients would welcome an opportunity to discuss and share their history with a radiologist.

After an internal discussion involving all stakeholders, covering all aspects of the examination process, and respecting spatial conditions, we decided to introduce a short medical history interview for MRI patients prior to the exam. We chose the modality because of a convenient proximity of the diagnostic workstations and the tomographs and because of a manageable number of patients.

Approximately one year after the first round of surveying the mandatory implementation of a medical history interview with our MRI-patients was established. The radiologists received a “medical history questionnaire”, which provided structure for the interview and included a check box about the need to change the originally scripted MRI protocol. The fraction of protocols that were adapted due to information from the interview was taken as a medical quality indicator. Independently, we repeated the survey amongst some MRI-patients. In the following year, we conducted the survey again amongst ultrasound-patients and one group of MRI-patients receiving a medical interview and another group of MRI-patients who had no interview.

Strikingly, over the years there was a remarkably constant appraisal of our services. Satisfaction with the friendliness and competence of our staff, waiting times, as well as potential recommendation and general satisfaction did not vary throughout the survey period and were independent of the examination modality. Likewise, modality-specific deviations or the marred attitude of patients with long waiting times prevailed over the years.

Only when the process changed, patients gave the respective feedback. As expected from the scrutinized first-year survey results, patients appreciated the attention by radiologists. What was surprising, however, was the extent of patient value achieved by a medical history interview lasting for approximately 3 minutes. Approval rating achieved levels typical of examinations requiring constant, ca. 30-minute care by a radiologist.

We interpret this result to mean that it is the mere existence and the resulting quality of radiologist-to-patient contact that increases patient satisfaction to a quantitatively maximal level. In other words, 3 minutes patient care are as effective in achieving patient satisfaction as a 30-minute close encounter during an ultrasound examination.

### Limitations

Our work may be seen in the context of ample of literature on the importance of patient satisfaction in radiology and its economic value [25], thereby also exposing its limitations:

There are many possibilities where radiologists could interact and communicate with their patients and create trust. We have chosen a way that fits into the constraints imposed by a high-throughput hospital and believe it is generally applicable. Our fundamental hypothesis is that the mere possibility to get sympathetic personal attention of a physician increases patient satisfaction. This would imply that interactions ranging from low-threshold handshakes to communication of diagnoses would have similar impacts. However, there are more dimensions beyond the scope of this study to the abstract value of “satisfaction” and the reduction to a sexpartite scale may not be an adequate tool to cover the whole spectrum. In this context, it would be interesting to validate the quality of the radiologist-patient communication, e.g. by differentiating between verbal, non-verbal communication, and clinical empathy.

We only evaluated patient feedback in a single institution and hence we cannot completely exclude that variable local practice patterns and traditions could influence the impression of medical interviews on patients. However, the Bernese might be a little bit slower, but they are not fundamentally different. In this respect, we would encourage other clinics to conduct similar initiatives in order to understand patient experiences better in their own setting.

In addition, our evaluation of the patient experience largely related to experiences occurring during the patient’s time in the radiology department. The total patient experience may be considered more widely to encompass a broader spectrum of contexts, ranging from the time when the examination is first ordered, to the referring physician receiving the results and taking the appropriate action for the patient.

Further limitations are the missing information of age/sex of the included patients and that though we explored associations between various themes and patient satisfaction, these were not associated with downstream health outcomes.

## Conclusion

Based on our findings we suggest implementing medical history interviews as a better alternative to “meet and greet” style “Radiology Cares” encounters, because interviews have a specific, additional, and beneficial effect on the conformity of the examination protocol. Thus, a medical interview increases patient value by affecting both patient satisfaction and the planning quality of radiologic examinations.

## Supporting information

S1 Table Overall satisfaction and recommendations is higher in patients waiting shorter. (DOCX)

S2 Table Overall satisfaction and recommendations is not different in in-patients and out-patients. (DOCX)

S3 Table Results from the initial survey demonstrating that ultrasound patients are responding differently than MRI patients. (DOCX)

S4 Table Motivations of ultrasound patients, who did not grade the contact with radiologists maximally. (DOCX)

S5 Table MRI patients having had the opportunity of a medical history interview had a significantly better impression of the radiologists. (DOCX)

S6 Table MRI patients having had the opportunity of a medical history interview rated contact with radiologists like ultrasound patients. (DOCX)

S7 Table Combined data of three rounds of surveys over three years. Satisfaction with radiologists was significantly higher amongst patients who had a personal contact.
(DOCX)

S8 Table Combined data of three rounds of surveys over three years comparing ultrasound patients and MRI patients with and without contact. Satisfaction with radiologists was significantly higher amongst patients who had a personal contact.
(DOCX)

S1 Fig Scheduling and schematic representation of the experimental procedure. (PDF)

S2 Fig Original questionnaire. (PDF)
