# Supplementary material for: Enhancing patient value efficiently: Medical history interviews create patient satisfaction and contribute to an improved quality of radiologic examinations
Source: PLoS One. 2018 Sep 26;13(9):e0203807. doi: 10.1371/journal.pone.0203807 (PMC6157877; doi:10.1371/journal.pone.0203807)
Supplement: S1 Table — (DOCX) [file pone.0203807.s001.docx]

**S1 Table:** **Overall satisfaction and recommendations is higher in patients waiting shorter.** Pairwise comparison of average overall satisfaction and recommendation of our radiology services dependent on subjective waiting times (less than 15, 15 to 30, or more than 30 minutes). Combined survey data of three years are presented. Data are expressed as the percentage of positive grading including a 95% confidence interval. Significances are calculated for the distribution of positive (6, 5, 4) versus negative (3, 2, 1) grading. Significances at the 99% confidence level or higher are marked in bold, significances at the 95% confidence level are in italic. For exact phrasing of questions refer to Table 1.

|  | positive grading (6, 5, 4) in % of answered questions (95% Wilson confidence interval) | | P value (chi square test) |
| --- | --- | --- | --- |
| question | >30 min | <15 min |  |
| 14  (service overall) | 95.65% (85.46-98.80) | 99.37% (98.52-99.72) | **0.007** |
| 15  (recommendation) | 93.48% (82.50-97.76) | 99.38% (98.54-99.73) | **<0.001** |
| number | (46) | (811) |  |
|  | Grading (%, cI) | |  |
| question | 15-30 min | <15 min |  |
| 14  (service overall) | 97.52% (92.96-99.15) | 99.37% (98.52-99.72) | *0.042* |
| 15  (recommendation) | 99.17% (95.43-99.85) | 99.38% (98.54-99.73) | 0.791 |
| number | (123) | (811) |  |
|  | Grading (%, cI) | |  |
| question | 15-30 min | >30 min |  |
| 14  (service overall) | 97.52% (92.96-99.15) | 95.65% (85.46-98.80) | 0.527 |
| 15  (recommendation) | 99.17% (95.43-99.85) | 93.48% (82.50-97.76) | *0.032* |
| number | (123) | (46) |  |
