# Supplementary material for: Enhancing patient value efficiently: Medical history interviews create patient satisfaction and contribute to an improved quality of radiologic examinations
Source: PLoS One. 2018 Sep 26;13(9):e0203807. doi: 10.1371/journal.pone.0203807 (PMC6157877; doi:10.1371/journal.pone.0203807)
Supplement: S2 Table — (DOCX) [file pone.0203807.s002.docx]

**S2 Table:** **Overall satisfaction and recommendations is not different in in-patients and out-patients.** Pairwise comparison of overall satisfaction and recommendation of our radiology services dependent on the type of stay (in-patient or out-patient). Combined survey data are presented. Data are expressed as the percentage of positive grading including a 95% confidence interval. Significances are calculated for the distribution of positive (6, 5, 4) versus negative (3, 2, 1) grading. Significances at the 99% confidence level or higher are marked in bold, significances at the 95% confidence level are in italic. For phrasing of questions refer to Table 1. Differences in satisfaction between in-patients and out-patients in questions 5, 9, 11, 12, and 13 are due to another mix of examination modalities with diverging levels of comfort and attention by radiologists.

|  | positive grading (6, 5, 4) in % of answered questions (95% Wilson confidence interval) | | | left blank in % of number of surveys | | |
| --- | --- | --- | --- | --- | --- | --- |
| question | in-patient | out-patient | P-values (chi square test) | in-patient | out-patient | P-values (chi square test) |
| 4a | 100% | 99.5% (98.7-99.8) | 0.287 | 0.5% | 0.3% | 0.636 |
| 4b | 99.5% (97.3-99.9) | 99.6% (98.8-99.9) | 0.885 | 5.6% | 5.5% | 0.972 |
| 5 | 95.4% (90.4-97.9) | 97.1% (95.6-98.1) | 0.314 | 39.1% | 9.3% | **<0.001** |
| 6 | 89.8% (84-6-93.3) | 90.2% (87.7-92.2) | 0.879 | 13.5% | 10.5% | 0.221 |
| 7 | 94.6% (90.6-97.0) | 92.5% (90.3-94.2) | 0.290 | 4.7% | 5.5% | 0.617 |
| 8a | 100% | 99.5% (98.7-99.8) | 0.288 | 0.9% | 0.4% | 0.331 |
| 8b | 100% | 99.6% (98.8-99.9) | 0.354 | 5.1% | 6.2% | 0.562 |
| 9 | 51.1% (43.9-58.3) | 59.3% (55.6-62.9) | *0.046* | 15.3% | 7.4% | **<0.001** |
| 10 | 99.5% (97.3-99.1) | 98.1% (96.8-98.9) | 0.148 | 3.7% | 3.5% | 0.904 |
| 11 | 97.1% (93.9-98.7) | 88.4% (85.9-90.5) | **<0.001** | 3.3% | 1.3% | 0.055 |
| 12 | 99.5% (97.3-99.9) | 98.8% (97.7-99.4) | 0.375 | 4.2% | 0.9% | **0.001** |
| 13 | 81.2% (74.6-86.4) | 93.5% (91.3-95.1) | **<0.001** | 1.0% | 6.5% | **0.001** |
| 14 | 99.0% (96.6-99.7) | 99.2% (98.3-99.6) | 0.822 | 3.3% | 1.6% | 0.116 |
| 15 | 99.5% (97.4-99.9) | 99.2% (98.3-99.6) | 0.613 | 0.5% | 1.4% | 0.249 |
| number | (215) | (761) |  |  |  |  |
|  | percentage of in-patients examined by modality | percentage of out-patients examined by modality |  |  |  |  |
| MRT | 4.2% | 56.4% |  |  |  |  |
| CT | 11.6% | 15.0% |  |  |  |  |
| X-ray | 58.1% | 7.6% |  |  |  |  |
| Ultrasound | 18.1% | 17.3% |  |  |  |  |
