# Supplementary material for: Enhancing patient value efficiently: Medical history interviews create patient satisfaction and contribute to an improved quality of radiologic examinations
Source: PLoS One. 2018 Sep 26;13(9):e0203807. doi: 10.1371/journal.pone.0203807 (PMC6157877; doi:10.1371/journal.pone.0203807)
Supplement: S3 Table — (DOCX) [file pone.0203807.s003.docx]

**S3 Table:** **Results from the initial survey demonstrating that ultrasound patients are responding differently than MRI patients.** Initial survey responses by ultrasound and MRI patients. Data are expressed as the percentage of positive grading including a 95% confidence interval and as the percentage of answered questions. Significances are calculated for the distribution of positive (6, 5, 4) versus negative (3, 2, 1) grading and for answered versus left blank questions. Significances at the 99% confidence level or higher are marked in bold, significances at the 95% confidence level are in italic. Ultrasound patients experiencing contact with radiologists have a significantly higher response rate at questions 9 and 13. On the other hand, they very frequently did not respond to question 5 about an MRI safety questionnaire. For exact phrasing of questions refer to Table 1.

|  | positive grading (6, 5, 4) in % of answered questions (95% Wilson confidence interval) | | | left blank in % of number of surveys | | |
| --- | --- | --- | --- | --- | --- | --- |
| question | MRI | Ultrasound | P-values (chi square test) | MRI | Ultrasound | P-values (chi square |
| 4a | 98.8% (95.6-99.7) | 100% (94.2-100) | 0.378 | 0.6% | 0% | 0.535 |
| 4b | 98.7%(95.3-99.6) | 100% (93.8-100) | 0.380 | 6.2% | 5.6% | 0.939 |
| 5 | 96.8% (92.7-98.6) | 93.5% (82.5-97.8) | 0.311 | 3.7% | 25.8% | **<0.001** |
| 6 | 90.2% (84.4-94.0) | 92.0% (81.2-96.9) | 0.704 | 5.6% | 19.4% | **0.002** |
| 7 | 89.5% (83.7-93.5) | 94.6% (85.2-98.1) | 0.269 | 5.6% | 11.3% | 0.136 |
| 8a | 99.4% (96.6-99. 9) | 100% (94.2-100) | 0.534 | 0.6% | 0% | 0.535 |
| 8b | 99.3% (96.4-99.9) | 100% (93. 9-100) | 0.532 | 6.2% | 4.8% | 0.702 |
| 9 | 28.0% (21.4-35.7) | 83.9% (72.8-91.0) | **<0.001** | 7.4% | 0% | *0.028* |
| 10 | 98.1% (94.6-99.4) | 93.6% (84.6-97.5) | 0.082 | 1.9% | 0% | 0.281 |
| 11 | 83.3% (76.7-88.4) | 96.8% (89.0-99.1) | **0.007** | 3.7% | 0% | 0.125 |
| 12 | 98.75% (95. 6-99.7) | 98.3% (91.0-99.7) | 0.802 | 1.2% | 4.8% | 0.102 |
| 13 | 86.2% (79.9-91.6) | 95.1% (86.5-98.3) | 0.083 | 20.4% | 1.6% | **<0.001** |
| 14 | 98.73% (95.5-99.7) | 98.4% (91.4-99.7) | 0.846 | 3.1% | 0% | 0.162 |
| 15 | 99.4% (96.5-99.9) | 100% (94.1-100) | 0.533 | 2.5% | 1.6% | 0.698 |
| number | (162) | (62) |  |  |  |  |
