# Supplementary material for: Enhancing patient value efficiently: Medical history interviews create patient satisfaction and contribute to an improved quality of radiologic examinations
Source: PLoS One. 2018 Sep 26;13(9):e0203807. doi: 10.1371/journal.pone.0203807 (PMC6157877; doi:10.1371/journal.pone.0203807)
Supplement: S4 Table — (DOCX) [file pone.0203807.s004.docx]

**S4 Table:** **Motivations of ultrasound patients, who did not grade the contact with radiologists maximally.** Results of a structured survey amongst 38 ultrasound patients to elucidate the reason of the high variability of question 9 (Have you had contact with a physician at this clinic, either before, during, or after the scan?).

|  | **reason** | **Absolute values and proportions (%)** |
| --- | --- | --- |
| grading 6 | No further interview | 16 (42%) |
| grading 5 to 1 | Did indeed grade the performance of the radiologists | 2 (5%) |
|  | Confused about role of investigator, about the location, or about the question | 10 (26%) |
|  | Estimated the number of medical doctors they have seen | 10 (26%) |
| sum |  | 38 |
