# Supplementary material for: Enhancing patient value efficiently: Medical history interviews create patient satisfaction and contribute to an improved quality of radiologic examinations
Source: PLoS One. 2018 Sep 26;13(9):e0203807. doi: 10.1371/journal.pone.0203807 (PMC6157877; doi:10.1371/journal.pone.0203807)
Supplement: S5 Table — (DOCX) [file pone.0203807.s005.docx]

**S5 Table: MRI patients having had the opportunity of a medical history interview had a significantly better impression of the radiologists.** Responses of MRI patients during the initial survey (without interview) and a year later, when 86% had a medical history interview. Data are expressed as the percentage of positive grading including a 95% confidence interval and as the percentage of answered questions. Significances were calculated for the distribution of positive (6, 5, 4) versus negative (3, 2, 1) grading and for answered versus left blank questions. Significances at the 99% confidence level or higher are marked in bold. Patients experiencing contact with radiologist rate questions 9 and 13 significantly higher and respond to question 13 more often. For exact phrasing of questions refer to Table 1.

|  | positive grading (6, 5, 4) in % of answered questions (95% Wilson confidence interval) | | | left blank in % of number of surveys | | |
| --- | --- | --- | --- | --- | --- | --- |
| question | without patient interview | with patient interview | P-values (chi square test) | without patient interview | with patient interview | P-values (chi square test) |
| 4a | 98.8% (95.6-99.7) | 98.9% (96.1-99.7) | 0.902 | 0.6% | 0% | 0.288 |
| 4b | 98.7% (95.3-99.6) | 99.4% (96.8-99.9) | 0.494 | 6.2% | 6.0% | 0.960 |
| 5 | 96.8% (92.7-98.6) | 97.8% (94.4-99.1) | 0.590 | 3.7% | 2.2% | 0.407 |
| 6 | 90.2% (84.5-94.0) | 92.2% (87.1-95.4) | 0.523 | 5.6% | 8.2% | 0.329 |
| 7 | 89.7% (83.7-93.5) | 92.0% (87.0-95.1) | 0.451 | 5.6% | 4.4% | 0.620 |
| 8a | 99.4% (96.6-99.9) | 99.4% (96.9-99.9) | 0.934 | 0.6% | 0.5% | 0.934 |
| 8b | 99.3% (96.4-99.9) | 98.8% (95.7-99.7) | 0.611 | 6.2% | 9.3% | 0.275 |
| 9 | 28.0% (21.4-35.7) | 80.6% (74.0-85.8) | **<0.001** | 7.4% | 6.6% | 0.767 |
| 10 | 98.1% (94.6-99.4) | 98.3% (95.1-99.4) | 0.895 | 1.9% | 2.7% | 0.582 |
| 11 | 83.3 (76.7-88.4) | 79.9% (73.4-85.1) | 0.418 | 3.7% | 1.6% | 0.233 |
| 12 | 98.8 (95.6-99.7) | 99.4% (96.9-99.9) | 0.494 | 1.2% | 1.1% | 0.907 |
| 13 | 86.8 (79.9-91.6) | 96.6 (92.8-98.4) | **0.001** | 20.4% | 3.3% | **<0.001** |
| 14 | 98.7 (95.5-99.6) | 98.9 (96.0-99.7) | 0.895 | 3.1% | 1.6% | 0.377 |
| 15 | 99.4 (96.5-99.9) | 97.8% (94.4-99.1) | 0.225 | 2.5% | 1.6% | 0.590 |
| number | (162) | (182) |  |  |  |  |
