# Supplementary material for: Enhancing patient value efficiently: Medical history interviews create patient satisfaction and contribute to an improved quality of radiologic examinations
Source: PLoS One. 2018 Sep 26;13(9):e0203807. doi: 10.1371/journal.pone.0203807 (PMC6157877; doi:10.1371/journal.pone.0203807)
Supplement: S6 Table — (DOCX) [file pone.0203807.s006.docx]

**S6 Table:** **MRI patients having had the opportunity of a medical history interview rated contact with radiologists like ultrasound patients.** Ultrasound patients during the initial survey and MRI patients in the subsequent year, when 86% had a medical history interview. Positive grading and response by first-year ultrasound patients and MRI patients in the second year (who had contact with physicians). Data are expressed as the percentage of positive grading including a 95% confidence interval and as the percentage of answered questions. Significances at the 99% confidence level or higher are marked in bold, significances at the 95% confidence level are in italic. Significant differences in the responses are due to the modality (questions 5 and 11). However, satisfaction with radiologists is indistinguishable (questions 9 and 13). For exact phrasing of questions refer to Table 1.

|  | positive grading (6, 5, 4) in % of answered questions (95% Wilson confidence interval) | | | left blank | | |
| --- | --- | --- | --- | --- | --- | --- |
| question | MRI | Ultrasound | P-values (chi square test) | MRI | Ultrasound | P-values (chi square test) |
| 4a | 98.9% (96.1-99.7) | 100% (94.2-100) | 0.407 | 0% | 0% |  |
| 4b | 99.4% (96.8-99.9) | 100% (93.8-100) | 0.559 | 6.0% | 5.6% | 0.908 |
| 5 | 97.8% (94.4 -99.1) | 93.5% (82.5-97.8) | 0.137 | 2.2% | 25.8% | **<0.001** |
| 6 | 92.2% (87.1-95.4) | 92.0% (81.2-96.9) | 0.960 | 8.2% | 19.4% | *0.016* |
| 7 | 92.0% (87.0-95.2) | 94.6% (85.2-98.1) | 0.523 | 4.4% | 11.3% | 0.051 |
| 8a | 99.5% (96.9-99.9) | 100% (94.2-100) | 0.558 | 0.5% | 0% | 0.559 |
| 8b | 98.8% (95.7-99.7) | 100% (93.9-100) | 0.396 | 9.3% | 4.8% | 0.264 |
| 9 | 80.6% (74.0-85.8) | 83.9% (72.8-91.0) | 0.569 | 6.6% | 0% | *0.038* |
| 10 | 98.3% (95.1-99.4) | 93.6% (84.6-97.5) | 0.056 | 2.7% | 0% | 0.187 |
| 11 | 79.9% (73.4-85.1) | 96.8% (89.0-99.1) | **0.002** | 1.6% | 0% | 0.309 |
| 12 | 99.4% (96.9-99.9) | 98.3% (91.0-99.7) | 0.404 | 1.1% | 4.8% | 0.073 |
| 13 | 96.6 (92.8-98.4) | 95.1% (86.5-98.3) | 0.595 | 3.3% | 1.6% | 0.493 |
| 14 | 98.9 (96.0-99.7) | 98.4% (91.4-99.7) | 0.762 | 1.6% | 0% | 0.309 |
| 15 | 97.8% (94.4-99.1) | 100% (94.1-100) | 0.239 | 1.6% | 1.6% | 0.985 |
| number | (182) | (62) |  |  |  |  |
