# Supplementary material for: Enhancing patient value efficiently: Medical history interviews create patient satisfaction and contribute to an improved quality of radiologic examinations
Source: PLoS One. 2018 Sep 26;13(9):e0203807. doi: 10.1371/journal.pone.0203807 (PMC6157877; doi:10.1371/journal.pone.0203807)
Supplement: S7 Table — Satisfaction with radiologists was significantly higher amongst patients who had a personal contact. (DOCX) [file pone.0203807.s007.docx]

**S7 Table:** **Combined data of three rounds of surveys over three years. Satisfaction with radiologists was significantly higher amongst patients who had a personal contact.** Positive grading and response by patients experiencing contact with physicians (ultrasound, MRI in year 2, first MRI group in year 3) or not (X-ray, CT, MRI in year 1, second MRI group in year 3). Data are expressed as the percentage of positive grading including a 95% confidence interval and as the percentage of answered questions. Significances at the 99% confidence level or higher are marked in bold. Satisfaction with radiologists is significantly better in the interview group (questions 9 and 13). For exact phrasing of questions refer to Table 1.

|  | positive grading (6, 5, 4) in % of answered questions (95% Wilson confidence interval) | | | left blank | | |
| --- | --- | --- | --- | --- | --- | --- |
| question | without contact | with contact | P-values (chi square test) | without contact | with contact | P-values (chi square test) |
| 4a | 99.6% (98.7-99.9) | 99.5% (98.3-99.9) | 0.786 | 0.7% | 0.0% | 0.081 |
| 4b | 99.4% (98.3-99.8) | 99.7% (98.6-100.0) | 0.456 | 6.4% | 5.1% | 0.383 |
| 5 | 96.6% (94.4-97.9) | 96.8% (94.4-98.1) | 0.872 | 20.0% | 10.2% | **<0.001** |
| 6 | 89.1% (86.0-91.6) | 90.7% (87.30-93.3) | 0.456 | 12.0% | 11.2% | 0.708 |
| 7 | 93.5% (91.1-95.4) | 92.3% (89.3-94.6) | 0.483 | 6.3% | 5.1% | 0.448 |
| 8a | 99.6% (98.7-99.9) | 99.5% (98.2-99.9) | 0.780 | 0.6% | 0.5% | 0.889 |
| 8b | 99.8% (98.9-100.0) | 99.5% (98.1-99.9) | 0.406 | 6.3% | 6.8% | 0.734 |
| 9 | 34.3% (30.1-38.8) | 82.1% (78.0-85.5) | **<0.001** | 13.6% | 3.9% | **<0.001** |
| 10 | 98.5% (97.0-99.2) | 98.0% (96.1-99.0) | 0.605 | 4.6% | 2.4% | 0.077 |
| 11 | 92.7% (90.1-94.6) | 87.5% (84.0-90.3) | **0.007** | 2.2% | 1.2% | 0.250 |
| 12 | 99.1% (97.8-99.6) | 98.8% (97.1-99.5) | 0.656 | 2.0% | 2.2% | 0.862 |
| 13 | 84.6% (80.7-87.8) | 96.7% (94.5-99.6) | **<0.001** | 27.2% | 3.2% | **<0.001** |
| 14 | 98.9% (97.5-99.5) | 99.0% (97.5-99.6) | 0.808 | 3.5% | 0.7% | **0.005** |
| 15 | 99.4% (98.4-99.8) | 98.5% (96.8-99.3) | 0.153 | 1.7% | 1.2% | 0.574 |
| number of patients | 544 | 412 |  |  |  |  |
