# Supplementary material for: Enhancing patient value efficiently: Medical history interviews create patient satisfaction and contribute to an improved quality of radiologic examinations
Source: PLoS One. 2018 Sep 26;13(9):e0203807. doi: 10.1371/journal.pone.0203807 (PMC6157877; doi:10.1371/journal.pone.0203807)
Supplement: S8 Table — Satisfaction with radiologists was significantly higher amongst patients who had a personal contact. (DOCX) [file pone.0203807.s008.docx]

**S8 Table:** **Combined data of three rounds of surveys over three years comparing ultrasound patients and MRI patients with and without contact. Satisfaction with radiologists was significantly higher amongst patients who had a personal contact.** Positive grading and response by patients experiencing contact with physicians (ultrasound, MRI in year 2, first MRI-group in year 3) or not (MRI in year 1, second MRI-group in year 3). Data are expressed as the percentage of positive grading including a 95% confidence interval and as the percentage of answered questions. Significances at the 99% confidence level or higher are marked in bold. Satisfaction with radiologists is significantly better in the interview group (questions 9 and 13). For exact phrasing of questions refer to Table 1.

|  | positive grading (6, 5, 4) in % of answered questions (95% Wilson confidence interval) | | | left blank | | | P-values (chi square test) | | | | | |
| --- | --- | --- | --- | --- | --- | --- | --- | --- | --- | --- | --- | --- |
| question | Ultrasound | MRI +contact | MRI  -contact | Ultrasound | MRI +contact | MRI  -contact | Positive (6,5,4) vs. negative (3,2,1) | Empty vs. answered | Positive (6,5,4) vs. negative (3,2,1) | Empty vs. answered | Positive (6,5,4) vs. negative (3,2,1) | Empty vs. answered |
|  |  |  |  |  |  |  | US vs. MRI +contact | | US vs. MRI -contact | | MRI +contact vs. MRI -contact | |
| 5 | 94.5% (89.5-97.2) | 98.2% (95.5-99.3) | 97.5% (94.3-98.9) | 20.8% | 1.7% | 3.8% | *0.047* | **<0.001** | 0.144 | **<0.001** | 0.611 | 0.183 |
| 9 | 85.7% (79.9-90.1) | 79.0% (73.0-83.9) | 29.7% (23.7-36.5) | 0.5% | 6.6% | 8.1% | 0.081 | **0.002** | **<0.001** | **<0.001** | **<0.001** | 0.525 |
| 11 | 95.6% (91.5-97.7) | 81.0% (75.3-85.6) | 84.2% (78.6-88.6) | 1.1% | 1.3% | 2.9% | **<0.001** | 0.841 | **<0.001** | 0.214 | 0.374 | 0.250 |
| 13 | 96.7% (93.0-98.5) | 96.8% (93.5-98.4) | 88.2% (82.3-92.3) | 1.1% | 4.8% | 23.0% | 0.954 | *0.032* | **0.003** | **<0.001** | **0.001** | **<0.001** |
| number of patients | 183 | 229 | 209 |  | | | | | | | | |
