# Supplementary material for: Enhancing patient value efficiently: Medical history interviews create patient satisfaction and contribute to an improved quality of radiologic examinations
Source: PLoS One. 2018 Sep 26;13(9):e0203807. doi: 10.1371/journal.pone.0203807 (PMC6157877; doi:10.1371/journal.pone.0203807)
Supplement: S1 Fig — (PDF) [file pone.0203807.s009.pdf]

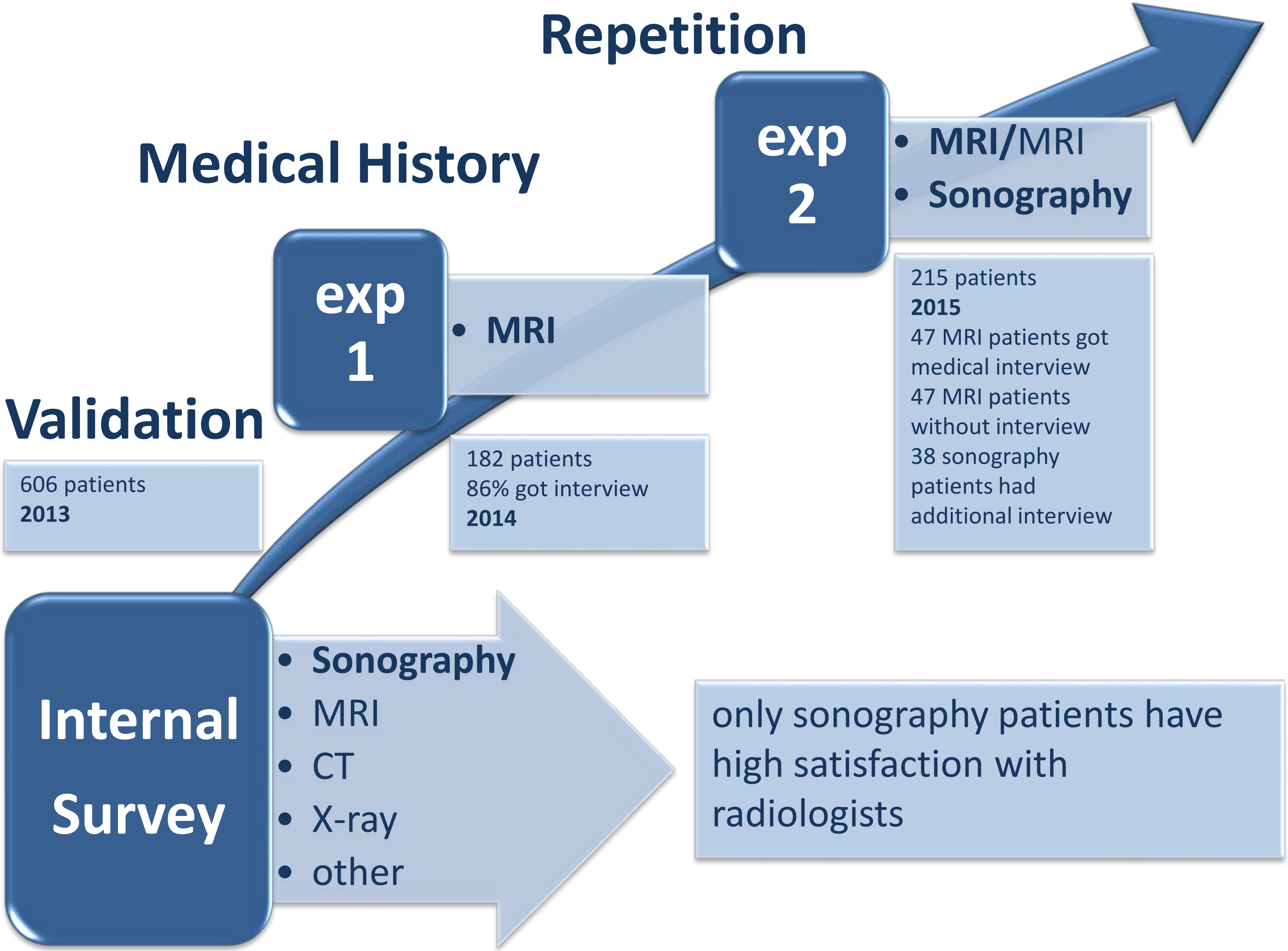

S1 Figure: Schematic Representation of the experimental procedure. A patient satisfaction survey demonstrated that patients with radiologist-contact (in bold) exhibit higher satisfaction. A year later, medical history interviews were introduced for (most of the) MRI-patients and 182 of them received the same questionnaire. In the following year, the survey was repeated amongst MRI patients who either had or had no medical history interview. Moreover, some of the sonography-patients had a personal, structured interview about their motivation concerning question 9.
