# Supplementary material for: Enhancing patient value efficiently: Medical history interviews create patient satisfaction and contribute to an improved quality of radiologic examinations
Source: PLoS One. 2018 Sep 26;13(9):e0203807. doi: 10.1371/journal.pone.0203807 (PMC6157877; doi:10.1371/journal.pone.0203807)
Supplement: S2 Fig — (PDF) [file pone.0203807.s010.pdf]

INSELSPITAL
UNIVERSITÄTSSPITAL BERN
HOPITAL UNIVERSITAIRE DE BERNE
BERN UNIVERSITY HOSPITAL

Patientenumfrage

Ihre Meinung ist uns wichtig…

Sehr geehrte Patientinnen und Patienten

Wir vom Radiologischen Institut des Inselspitals sind als ärztlicher Dienstleister bemüht, Ihnen Diagnostik auf Spitzenniveau zukommen zu lassen und Ihnen Ihre Untersuchung so angenehm wie möglich zu gestalten.

Um diesem Anspruch gerecht zu werden, benötigen wir Ihre Mithilfe und Ihre konstruktive Kritik. Daher möchte ich Sie bitten, sich kurz Zeit zu nehmen und diesen Fragebogen auszufüllen. Die Befragung erfolgt anonym und hat keinerlei Auswirkungen auf Ihre Behandlung, aber sie hilft uns, unsere Dienstleistung für künftige Patienten zu verbessern.

Ich hoffe, wir haben Ihre Erwartungen erfüllt und ich möchte mich sehr herzlich für Ihre Rückmeldung bedanken.

Prof. Dr. Dr. Johannes Heverhagen

Bitte füllen Sie den Fragebogen möglichst vollständig aus und werfen sie ihn danach in den markierten Briefkasten im Wartebereich.

Die ersten zwei Fragen betreffen Ihre Untersuchungsmethode und können entsprechend ausgewählt werden. Die restlichen Fragen folgen einer sechsstufigen Bewertungsskala, die von einer sehr guten Bewertung ganz links bis zu einer sehr schlechten Bewertung ganz rechts reicht.
Bitte kreuzen Sie das Kästchen an, das Ihre persönliche Einschätzung und Erfahrung am besten beschreibt.

sehr gut ❑ ❑ ❑ ❑ ❑ ❑ sehr schlecht

Sie können uns gerne noch eigene Verbesserungsvorschläge unterbreiten.

Inselspital, Universitätsinstitut für Diagnostische, Interventionelle und Pädiatrische Radiologie (DIPR), Freiburgstrasse 10, 3010 Bern
Departement Radiologie, Neuroradiologie und Nuklearmedizin, Prof. Dr. Dr. Johannes Heverhagen, Direktor und Chefarzt
Telefon: +41 (0)31 632 24 35, Fax: +41 (0)31 632 48 74, E-Mail: Johannes.Heverhagen@insel.ch, www.radiologie.insel.ch/de

Bern, den ____________________

1. Wie wurde Ihre Untersuchung durchgeführt? ❑ ambulant ❑ stationär

2. Welche Untersuchung wurde bei Ihnen durchgeführt?
❑ Magnetresonanztomographie
❑ Computertomographie
❑ Angiographie
❑ Mammographie
❑ Röntgen (Skelett, Lunge)
❑ Durchleuchtung
❑ Ultraschall

3. Wie lange mussten Sie warten? ❑ <15 min ❑ 15-30 min ❑ >30 min

4. Wie wurden Sie empfangen?
a) Freundlichkeit — sehr gut ❑❑❑❑❑❑ sehr schlecht
b) Kompetenz — sehr gut ❑❑❑❑❑❑ sehr schlecht

5. Wurden Sie bezüglich des Gesundheitsfragebogens ausreichend informiert? — sehr gut ❑❑❑❑❑❑ sehr schlecht

6. Wurden Sie über die Wartezeit informiert? — sehr gut ❑❑❑❑❑❑ sehr schlecht

7. Wie empfanden Sie die Wartezeit? — sehr kurz ❑❑❑❑❑❑ sehr lange

8. Wie wurden Sie vom radiologisch-technischen Personal betreut?
a) Freundlichkeit — sehr gut ❑❑❑❑❑❑ sehr schlecht
b) Kompetenz — sehr gut ❑❑❑❑❑❑ sehr schlecht

9. Hatten Sie hier am Radiologischen Institut vor, während oder nach der Untersuchung Kontakt mit einem Arzt oder Ärztin? — sehr viel ❑❑❑❑❑❑ gar keinen

10. Wurde auf Ihre Befindlichkeit vor und während der Untersuchung Rücksicht genommen? — sehr gut ❑❑❑❑❑❑ sehr schlecht

11. Wie empfanden Sie die Untersuchung selbst? — sehr angenehm ❑❑❑❑❑❑ sehr unangenehm

12. Wurden Sie nach der Untersuchung gut betreut und verabschiedet? — sehr gut ❑❑❑❑❑❑ sehr schlecht

13. Wie bewerten Sie die ärztliche Betreuung? — sehr gut ❑❑❑❑❑❑ sehr schlecht

14. Wie beurteilen Sie die radiologische Dienstleistung insgesamt? — sehr gut ❑❑❑❑❑❑ sehr schlecht

15. Können Sie Ihren Freunden und Verwandten das Radiologische Institut weiterempfehlen? — auf jeden Fall ❑❑❑❑❑❑ gar nicht

Anmerkungen und Anregungen zur Verbesserung unserer Dienstleistung:

____

____

____
